# Prevalence and associated factors of eating disorders among metabolic bariatric surgery patients in Egypt

**DOI:** 10.1186/s40337-025-01373-0

**Published:** 2025-08-25

**Authors:** Mohamed Hany, Hagar Ahmad Aly Yassin, Asmaa Hamdy, Ehab Elmongui, Anwar Ashraf Abouelnasr, Bart Torensma

**Affiliations:** 1 Department of Surgery, Medical Research Institute, Alexandria University, 165 Horreya Avenue, Hadara, 21561 Alexandria Egypt; 2 High Institute of Public Health, Alexandria University, Alexandria, Egypt; 3 Clinical Epidemiology, Erasmus MC, Rotterdam, the Netherlands

**Keywords:** Eating disorders, Binge eating disorder, Night eating syndrome, Bulimia nervosa, Metabolic bariatric surgery, Obesity and mental health, Psychiatric in associated medical problems, Bariatric surgery patients, Prevalence of eating disorders, Risk factors for eating disorders

## Abstract

**Background:**

Obesity is a growing global epidemic associated with significant morbidity and mortality. Metabolic bariatric surgery (MBS) is an effective intervention for obesity and its associated medical problems. However, eating disorders (EDs) are prevalent among MBS patients and may influence postoperative weight loss outcomes. Despite the well-documented impact of EDs in Western populations, data on their prevalence and associated factors among MBS patients in the Middle East and North Africa (MENA) region remain scarce. This study aims to determine the prevalence of EDs and identify their associated factors in a large cohort of Egyptian MBS patients.

**Methods:**

A cross-sectional study was conducted at the Bariatric Surgery Center of Madina Women’s Hospital in Alexandria, Egypt, including all adult patients for MBS from August 2022 to November 2024 (*N* = 3,240). A psychiatrist conducted structured clinical interviews based on the DSM-5 criteria using the Structured Clinical Interview for DSM-5 (SCID-5-RV) to diagnose EDs, including binge eating disorder (BED), bulimia nervosa (BN), and night eating syndrome (NES). Logistic regression analyses were performed to assess factors associated with E.Ds.

**Results:**

The overall prevalence of EDs in the study cohort was 47.8%, with BED being the most common disorder (36.1%), followed by NES (22.8%) and BN (2.1%). Female gender was significantly associated with a higher risk of BED OR 1.68, 95% CI: 1.38–2.04, *p* < 0.001). Heavy smoking was associated with significantly lower odds of BED compared to non-smoking (OR = 0.44, 95% CI: 0.30–0.63, *p* < 0.001), suggesting a potential protective association, but increased associated with NES (OR = 2.04, 95% CI: 1.42–2.90, *p* < 0.001). Psychiatric in associated medical problems, particularly depressive disorders (OR = 1.74 for BED, OR = 1.35 for NES, *p* < 0.05) and borderline personality disorder (OR = 1.56 for BED, OR = 1.91 for NES, *p* < 0.05), were significantly associated with increased ED risk.

**Conclusions:**

EDs are highly prevalent among MBS patients in Egypt, with BED being the most common. Female gender, younger age, psychiatric disorders, and smoking habits are key predictors of E.Ds. Given the potential impact of EDs on postoperative outcomes, routine psychiatric screening is essential for preoperative assessment. Future research should explore the long-term effects of EDs on weight loss and surgical outcomes.

**Trial registration:**

Not applicable (observational study).

## Abstract (plain language summary)

Obesity is a significant health concern worldwide, prompting many individuals to consider metabolic bariatric surgery (MBS) as an effective treatment option. However, the presence of eating disorders (EDs) among those seeking MBS can influence the success of the surgery and overall health outcomes. While studies in Western countries have explored this issue, there is limited information about the prevalence of EDs and associated factors among MBS patients in the Middle East and North Africa (MENA) region.

Our study examined 3,240 adults in Egypt who were preparing for the MBS between August 2022 and November 2024. We found that nearly half (47.8%) had an ED, with binge eating disorder (BED) being the most common (36.1%), followed by night eating syndrome (NES) (22.8%), and bulimia nervosa (BN) (2.1%). Women were more likely to have BED, and younger individuals were generally more prone to EDs. Interestingly, heavy smoking was associated with a lower risk of BED but a higher risk of NES. Additionally, those with mental health conditions like depression or borderline personality disorder had a greater likelihood of having an ED.

These findings emphasize the importance of routine mental health screenings for individuals considering MBS, particularly in regions like Egypt where such data has been limited. Understanding and addressing EDs before surgery can lead to better health outcomes and enhance the overall effectiveness of MBS.

## Background

Obesity is a growing global health epidemic with substantial public health and economic implications that continues to rise unacceptably [1]. As a significant risk factor for chronic diseases, including cardiovascular disease, stroke, cancer, respiratory disorders, and diabetes, obesity contributes substantially to increased morbidity and mortality globally [2–5]. If current trends persist, the economic burden of obesity is expected to reach $4.32 trillion annually by 2035, accounting for approximately 3% of the global gross domestic product [6].

The prevalence of obesity demonstrates significant regional disparities, with particularly elevated rates observed in the Middle East and North Africa (MENA), Central and Eastern Europe, and North America. In Egypt specifically, data from the 2019 national health survey reveal that the prevalence of adult obesity reaches 39.8%, affecting approximately 18.7 million adults. This substantial disease burden translates to a considerable economic impact, quantified at 62 billion Egyptian pounds annually, placing significant strain on the nation’s healthcare infrastructure [7].

Obesity treatments have included lifestyle, pharmacological, and surgical interventions. Generally, a multifactorial approach to treating obesity is most effective [8]. Lifestyle interventions have shown short-term reductions in weight, while newer pharmacological approaches, particularly the development of incretin-based therapies such as Glucagon-like peptide-1 agonists, have received increasing support for their efficacy [9]. However, the use of this class of medications is limited in many patients due to their elevated cost, patients’ noncompliance, and potential adverse events [10, 11].

Metabolic bariatric surgery (MBS) represents a well-established and effective intervention for obesity, offering substantial and sustained weight loss, as well as improvements in associated medical conditions [12, 13]. However, postoperative weight outcomes can be influenced by several factors, including maladaptive eating, lifestyle behaviors, psychologically associated medical problems, and eating disorders (EDs) [14, 15]. Understanding these variables is crucial for optimizing patient outcomes and tailoring treatment approaches in MBS medicine [14].

EDs affect up to 8.4% of women and 2.2% of men worldwide [16]. In the Arab world, ED prevalence is estimated at 3.2%, though it may be underestimated due to cultural stigma and the evolving understanding of EDs in the region [16, 17]. Among adults seeking treatment for obesity worldwide, EDs have notable prevalence, with binge eating disorder (BED) representing the most common subtype [18].

Among MBS patients, EDs are common; studies have reported varying prevalence rates, with 28.5% of Italian and 32% of Brazilian MBS patients diagnosed with EDs [19, 20]. EDs can persist or redevelop postoperatively, negatively affecting weight loss outcomes and leading to recurrent weight gain (RWG) [21–24]. Recent clinical guidelines emphasized that the primary goal in managing patients with co-occurring obesity and EDs should be the psychological treatment of the ED, as the weight loss interventions may exacerbate eating pathology [25].

A recent systematic review has revealed the elevated prevalence of BED among bariatric surgery candidates before surgery, reaching 50% in some studies. Following surgery, the prevalence of BED significantly decreases in the short-term follow-up, but at longer-term follow-up (36 months and beyond), binge eating symptoms may re-emerge and, in some cases, return to pre-surgery levels, leading to less favorable psychological and weight loss outcomes post-surgery [8].

However, data on the prevalence and associated factors of EDs in MBS patients from the MENA region remain scarce. Applying findings from Western studies to MENA populations may not be valid, since EDs are strongly shaped by sociocultural factors that vary significantly between regions [26]. Factors such as media exposure, family dynamics, and cultural ideals significantly influence body image perceptions and contribute to the development of disordered eating behaviors [27, 28]. The primary objectives are to determine the prevalence of EDs among MBS candidates and identify associated risk factors among them.

## Methods

### Study design and setting

This cross-sectional study was conducted at the Madina Women’s Hospital Bariatric Surgery Center in Alexandria, Egypt. The study was conducted in accordance with the principles of the Declaration of Helsinki and was approved by the Ethics Committee Board of the Medical Research Institute, Alexandria University, with approval reference number IORG0008812; E/C.S/N.R4/2022. Before enrollment in the study, written informed consent was obtained from all patients.

### Inclusion and exclusion criteria

The study included adult patients who had undergone MBS between August 20, 2022, and November 13, 2024, with a BMI greater than 40 kg/m² or a BMI greater than 35 kg/m² and medically associated problems; no exclusion criteria were applied for this study.

### Data collection

Each patient underwent a structured clinical interview conducted by a psychiatrist consultant. Demographic data, including age, gender, and marital status, were recorded. Anthropometric measurements, including height and weight, were obtained, and BMI was calculated. Smoking status and intensity were assessed. Medical history was obtained, including chronic medical conditions and previously diagnosed psychiatric disorders.

To assess eating disorders, all patients completed the Structured Clinical Interview for DSM-5 (Diagnostic and Statistical Manual of Mental Disorders, Fifth Edition) [29]. Research Version (SCID-5-RV) Module I [27] administered by a trained psychiatrist. Diagnoses were made according to DSM-5 criteria, including binge eating disorder, bulimia nervosa, anorexia nervosa, and night eating syndrome, which were categorized under other specified eating disorders.

### Statistical analysis

Descriptive and inferential statistics were performed. Categorical variables were presented as frequencies and percentages [n (%)], while the continuous variables—age and anthropometric measures (height, weight, and BMI)—were summarized as mean ± standard deviation (SD). Univariable and multivariable logistic regression analyses were conducted to examine factors associated with each eating disorder. Results are presented as odds ratios (ORs) for the univariable models and adjusted odds ratios (AORs) for the multivariable models, along with their corresponding 95% confidence intervals (CIs). To ensure stable model estimation, predictors were included only when there were at least 10 events of the eating disorder per variable. Statistical significance was set at *p* ≤ 0.05. All statistical analyses were performed using R software version 4.4.2.

### Sample size calculation

Sample size was calculated to estimate eating disorder prevalence with 95% confidence and 2% margin of error. Using the most conservative prevalence estimate (p = 0.5), the ‘epi.sssimpleestb’ function from the ‘epiR’ package in R determined a minimum required sample size of 2,401 patients. This calculation used the formula n = Z² × p × (1 - p) / E², where Z = 1.96, *p* = 0.5, and E = 0.02. All 3,240 eligible patients attending the bariatric surgery clinic during the study period were included to minimize selection bias [30, 31].

## Results

### Demographic and general characteristics

The study cohort consisted of 3,240 MBS patients, with 76.6% of them being female. The mean ± SD age of the patients was 36.8 ± 11.6 years. Most patients were married (65.6%), while 30.1% were single, 2.9% were divorced, and 1.5% were widowed. The mean body mass index (BMI) of the cohort was 45.2 ± 8.2 kg/m². Non-smoking was in 89.1% of patients, while 5.4% reported heavy smoking. The primary type of smoking was cigarettes in 10.5%. Substance use was rare, reported in 0.5% of patients, and alcohol consumption was observed in 0.2% of the cohort (Table 1).

**Table 1:** Demographics and general characteristics of the patients

Variable	*n* (%)
**Demographics**	
Age	36.8 ± 11.6
Gender	
Female	2481 (76.6)
Male	759 (23.4)
Marital Status	
Married	2125 (65.6)
Single	975 (30.1)
Divorced	93 (2.9)
Widow	47 (1.5)
**Anthropometrics**	
Weight	124.3 ± 25.3
Height	165.7 ± 8.9
BMI	45.2 ± 8.2

Cell values represent frequency and percentage n (%) for categorical variables and mean ± standard deviation (M ± SD) for continuous variables. BMI: Body Mass Index.

The study cohort demonstrated a high burden of obesity-related comorbidities, with obstructive sleep apnea being the most prevalent condition affecting nearly 60% of patients (*n* = 1,940, 59.9%), though only a small subset required CPAP therapy (*n* = 19, 0.6%). Musculoskeletal complications were common, with over one-third of patients experiencing osteoarthritis (*n* = 1,181, 36.5%) and chronic lung disease affecting nearly a quarter of the cohort (*n* = 746, 23.0%). Cardiovascular risk factors were prevalent, including hypertension in 22.3% (*n* = 722) and hypercholesterolemia in 9.8% (*n* = 319) of patients. Metabolic disorders were frequently observed, with diabetes present in 12.7% (*n* = 413) and hypothyroidism in 9.0% (*n* = 290) of the study population. Female-specific conditions were notably common, including menstrual disorders (18.3%, *n* = 592), menopause (9.2%, *n* = 297), and polycystic ovarian syndrome (7.3%, *n* = 236). Gastrointestinal comorbidities included GERD in 13.7% (*n* = 445) and inflammatory bowel disease in 6.9% (*n* = 224) of patients. Less frequent but clinically significant conditions included cardiac disease (3.6%, *n* = 117), peripheral vascular disease (3.0%, *n* = 96), and various other systemic conditions, reflecting the comprehensive health burden typically observed in patients seeking bariatric surgical intervention.

### Smoking

Smoking patterns in the study population revealed that the majority of participants were non-smokers, with 89.1% (*n* = 2,886) reporting no smoking history. Among those with smoking exposure, occasional smokers comprised 1.3% (*n* = 41) and former smokers represented 2.7% (*n* = 88) of the cohort. Smoking intensity varied among current smokers, with light smoking affecting 3.3% (*n* = 108), medium intensity smoking in 2.2% (*n* = 71), and heavy smoking observed in 5.4% (*n* = 175) of participants.

Traditional cigarettes were the most common form of tobacco use, utilized by 10.5% (*n* = 340) of patients, followed by shisha smoking in 6.2% (*n* = 201).

Modern smoking alternatives included vaping in 3.6% (*n* = 118) and IQOS heated tobacco products in 1.1% (*n* = 36) of the population. Substance use beyond tobacco was minimal, affecting only 0.5% (*n* = 17) of participants, while alcohol consumption was rare, reported by just 0.2% (*n* = 7) of the cohort. These low rates of substance use and alcohol consumption likely reflect cultural and religious influences prevalent in the Egyptian population, providing important context for understanding the behavioral risk profile of this bariatric surgery cohort.

### Prevalence of eating disorders

Eating disorders were identified in 47.8% of the patients. The most prevalent disorder was BED with 36.1% of the patients (95% CI: 34.4–37.8%). Night eating syndrome (NES) was observed in 22.8% of patients (95% CI: 21.4–24.2%), while bulimia nervosa (BN) had the lowest prevalence at 2.1% (95% CI: 1.6–2.6%).

### Psychiatric history

Psychiatric comorbidities were present in a substantial portion of the study population, with depressive disorders being the most prevalent mental health condition affecting 12.8% of patients (*n* = 416). Personality disorders, specifically borderline personality disorder, were diagnosed in 5.1% of the cohort (*n* = 164), while anxiety disorders affected 4.7% of patients (*n* = 151). Less common but clinically significant psychiatric conditions included schizophrenia and other psychotic disorders in 0.9% (*n* = 28) and bipolar disorder in 0.7% (*n* = 24) of participants. Psychopharmacological treatment patterns reflected the psychiatric burden, with antidepressants being the most frequently prescribed psychiatric medication, used by 8.7% of patients (*n* = 282). Anxiolytics were prescribed to 4.1% of the cohort (*n* = 132), while mood stabilizers were utilized in 1.2% (*n* = 38) and antipsychotics in 0.5% (*n* = 15) of patients. This psychiatric comorbidity profile underscores the complex mental health landscape among bariatric surgery candidates and highlights the importance of comprehensive psychiatric assessment and ongoing mental health support in this population.

### Factors associated with binge eating disorder

Unadjusted analysis:

Increasing age was associated with lower odds of BED (OR = 0.97 per year of age, 95% CI: 0.97–0.98, *p* < 0.001), meaning younger patients had relatively higher odds of experiencing BED. Females had significantly higher odds than males (OR = 1.79, 95% CI: 1.5–2.2, *p* < 0.001). Being divorced (OR = 1.57, 95% CI: 1.03–2.38, *p* = 0.035), single (OR = 1.41, 95% CI: 1.21–1.65, *p* < 0.001). Heavy smoking was associated with significantly lower odds of BED compared to non-smoking (OR = 0.44, 95% CI: 0.30–0.63, *p* < 0.001), suggesting a potential protective association. Psychiatric factors such as depressive disorder (OR = 1.83, 95% CI: 1.49–2.25, *p* < 0.001), anxiety disorder (OR = 1.48, 95% CI: 1.06–2.05, *p* = 0.020), and borderline personality disorder (OR = 1.78, 95% CI: 1.30–2.44, *p* < 0.001) were also associated (Table 2).

**Table 2:** Logistic regression analyses for the factors associated with eating disorders

Factor	Binge Eating Disorder		Night Eating Syndrome	
	OR (95% CI)	*p*	OR (95% CI)	*p*
**Age**	0.97 (0.97, 0.98)	< 0.001*	0.99 (0.99, 1.00)	0.079
**Gender**				
Male	Reference		Reference	
Female	1.79 (1.50, 2.15)	< 0.001*	1.01 (0.83, 1.23)	0.912
**Marital Status**				
Married	Reference		Reference	
Divorced	1.57 (1.03, 2.38)	0.035*	1.73 (1.10, 2.67)	0.016*
Single	1.41 (1.21, 1.65)	< 0.001*	0.99 (0.83, 1.19)	0.956
Widow	0.76 (0.38, 1.41)	0.406	1.32 (0.67, 2.46)	0.399
**Smoking intensity**				
None	Reference		Reference	
Light	1.12 (0.75, 1.65)	0.573	1.44 (0.93, 2.18)	0.094
Medium	0.62 (0.35, 1.03)	0.076	0.96 (0.52, 1.66)	0.882
Heavy	0.44 (0.30, 0.63)	< 0.001*	1.96 (1.41, 2.70)	< 0.001*
**Substance use**	1.24 (0.45, 3.24)	0.663	4.89 (1.87, 13.50)	0.001*
**Psychiatric illnesses**				
Depressive disorder	1.83 (1.49, 2.25)	< 0.001*	1.39 (1.10, 1.74)	0.006*
Anxiety disorder	1.48 (1.06, 2.05)	0.020*	1.19 (0.81, 1.71)	0.366
Borderline personality disorder	1.78 (1.30, 2.44)	< 0.001*	1.18 (0.81, 1.67)	0.381
Schizophrenia & other psychotic illnesses	0.98 (0.44, 2.10)	0.965	1.89 (0.84, 4.04)	0.108

Odds ratios (OR) and 95% confidence intervals (95% CI) were derived from univariable logistic regression models examining the association between each factor and the likelihood of binge eating disorder or night eating syndrome. Asterisk (*) indicates statistical significance at *p* < 0.05. For categorical variables, the term “Reference” denotes the baseline comparison group against which other categories were compared.

### Adjusted analysis

Potential confounders in the multivariable model showed several associations remained while others attenuated. Age (AOR = 0.97, 95% CI: 0.96–0.98, *p* < 0.001), female gender (AOR = 1.68, 95% CI: 1.38–2.04, *p* < 0.001), heavy smoking (AOR = 0.56, 95% CI: 0.37–0.83, *p* = 0.005), depressive disorder (AOR = 1.76, 95% CI: 1.40–2.21, *p* < 0.001), and borderline personality disorder (AOR = 1.54, 95% CI: 1.12–2.13, *p* = 0.008) remained independently associated with BED, while the associations with marital status and anxiety disorder were no longer statistically significant (Table 3).

**Table 3:** Multiple logistic regression analysis for the factors associated with eating disorders

Factor	Binge Eating Disorder		Night Eating Syndrome	
	AOR (95% CI)	*p*	AOR (95% CI)	*p*
**Constant**	1.83 (1.27, 2.63)	0.001*	0.45 (0.30, 0.67)	< 0.001*
**Age**	0.97 (0.96, 0.98)	< 0.001*	0.99 (0.98, 1.00)	0.009*
**Gender**				
**Male**	Reference		Reference	
**Female**	1.68 (1.38, 2.04)	< 0.001*	1.15 (0.93, 1.44)	0.199
**Marital Status**				
Married	Reference		Reference	
Divorced	1.33 (0.86, 2.04)	0.201	1.57 (0.99, 2.44)	0.051
Single	0.92 (0.75, 1.13)	0.405	0.81 (0.64, 1.02)	0.076
Widow	0.91 (0.45, 1.73)	0.787	1.40 (0.70, 2.66)	0.321
**Smoking intensity**				
None	Reference		Reference	
Light	1.13 (0.75, 1.69)	0.547	1.39 (0.89, 2.12)	0.138
Medium	0.68 (0.39, 1.17)	0.177	0.95 (0.51, 1.68)	0.870
Heavy	0.56 (0.37, 0.83)	0.005*	2.04 (1.42, 2.90)	< 0.001*
**Substance use**	2.15 (0.75, 5.89)	0.139	4.02 (1.49, 11.41)	0.006*
**Psychiatric illnesses**				
Depressive disorder	1.76 (1.40, 2.21)	< 0.001*	1.35 (1.04, 1.74)	0.020*
Anxiety disorder	1.01 (0.70, 1.46)	0.949	0.99 (0.65, 1.48)	0.959
Borderline personality disorder	1.54 (1.12, 2.13)	0.008*	1.19 (0.82, 1.70)	0.352
Schizophrenia & other psychotic illnesses	0.98 (0.43, 2.14)	0.965	1.91 (0.84, 4.14)	0.107

Adjusted odds ratios (AOR) and 95% confidence intervals (95% CI) were obtained from multivariable logistic regression models assessing independent associations between each factor and the likelihood of binge eating disorder or night eating syndrome. Asterisk (*) indicates statistical significance at *p* < 0.05. For categorical variables, “Reference” indicates the reference category used for comparison within each subgroup.

### Factors associated with night eating syndrome

#### Unadjusted analysis

For age (OR = 0.99, 95% CI: 0.99–1.00, *p* = 0.079), divorced status (OR = 1.73, 95% CI: 1.10–2.67, *p* = 0.016), heavy smoking (OR = 1.96, 95% CI: 1.41–2.70, *p* < 0.001), substance use (OR = 4.89, 95% CI: 1.87–13.50, *p* = 0.001), and depressive disorder (OR = 1.39, 95% CI: 1.10–1.74, *p* = 0.006) (Table 2).

#### Adjusted analysis

These associations were largely consistent with age (AOR = 0.99, 95% CI: 0.98–1.00, *p* = 0.009), heavy smoking (AOR = 2.04, 95% CI: 1.42–2.90, *p* < 0.001), substance use (AOR = 4.02, 95% CI: 1.49–11.41, *p* = 0.006), and depressive disorder (AOR = 1.35, 95% CI: 1.04–1.74, *p* = 0.020) maintaining their significance, while the association with marital status was attenuated (Table 3).

## Discussion

Eating disorders are increasingly recognized as significant factors influencing the outcomes of MBS. Their presence can contribute to postoperative challenges, including RWG and psychological distress [23, 24]. This study aimed to determine the prevalence and associated factors of eating disorders among MBS patients using the Structured Clinical Interview for DSM-5 (SCID-5) [29].

In our study, nearly half of the MBS patients (47.8%) met the diagnostic criteria for an eating disorder. BED was the most prevalent (36.1%), followed by NES (22.8%), while BN was the least common (2.1%). Psychiatric disorders were also common in our cohort, with 21.1% of patients reporting a history of mental health conditions, most frequently depression (12.8%). Our findings indicated that depression and borderline personality disorder may increase the odds of BED. At the same time, younger age, male gender, and heavy smoking were linked to a lower likelihood of BED. For NES, heavy smoking and depression were significant predictors, while younger patients had slightly lower odds of developing NES.

Our findings reveal a remarkably higher prevalence of eating disorders (47.8%) among Egyptian bariatric surgery candidates, with binge eating disorder being most prevalent at 36.1%. This substantially exceeds rates reported in recent international systematic reviews and meta-analyses. Taba et al. (2021) [32], in their comprehensive systematic review and meta-analysis of 19 studies encompassing 3,223 participants from multiple countries, reported an overall eating disorder prevalence of only 7.83% in the postoperative period, with binge eating disorder specifically affecting 3.81% of patients.

Similarly, the foundational work from the LABS consortium, particularly Mitchell et al.‘s landmark LABS-2 study involving 2,266 participants [33], reported a BED prevalence of 15.7% - still significantly lower than our findings. The LABS-3 data referenced by Heinberg et al. showed an even lower BED prevalence of 6.1% [34], establishing methodological standards for eating disorder assessment in bariatric populations.

Our substantially higher prevalence rates compared to these seminal US studies suggest important cultural, methodological, or population-specific factors operating within the MENA region that warrant further investigation. These disparities may reflect differences in cultural attitudes toward eating behaviors, varying levels of psychiatric comorbidity, distinct socioeconomic factors, or potentially different assessment timing relative to surgical intervention.

The relationship between preoperative eating disorders and metabolic bariatric surgery outcomes has been extensively studied, though with mixed findings that provide important context for our results. Kops et al. (2021) conducted a rigorous systematic review and meta-analysis of 19 studies including 3,223 participants (80.25% women, median age 41 years) specifically examining the association between preoperative binge eating and postoperative weight loss outcomes [35].

Their analysis revealed no significant differences in percentage total weight loss between BED and non-BED groups at multiple follow-up time points (6, 12, 24, 36, and 60 months), suggesting that preoperative binge eating may not necessarily compromise surgical success. However, the exceptionally high prevalence of eating disorders observed in our Egyptian cohort (47.8% overall, 36.1% BED) raises important questions about whether these findings from predominantly Western populations can be generalized to MENA region patients.

The theoretical framework established by Sarwer et al. provides crucial context [36], emphasizing that eating disorders exist within broader psychopathology patterns characterized by impulsivity and emotional dysregulation as shared constructs. Given our findings of significant associations between eating disorders and psychiatric comorbidities, particularly depressive disorders and borderline personality disorder, careful longitudinal monitoring of surgical outcomes in this high-risk population will be essential.

Recent evidence provides encouraging insights into the therapeutic relationship between metabolic bariatric surgery and eating disorder symptomatology that is particularly relevant given our high prevalence findings. Reche-García et al. (2024) conducted a comprehensive systematic review and meta-analysis of 22 studies involving 1,587 participants, examining the effectiveness of various interventions in reducing food addiction symptoms [37].

Their analysis of 15 studies suitable for meta-analysis demonstrated that metabolic bariatric surgical interventions showed the highest efficacy in improving food addiction symptoms (standardized mean difference = 1.17; 95% CI, 0.58–1.76), significantly outperforming both pharmacological (SMD = 1.11) and behavioral interventions (SMD = 0.48). This therapeutic potential of metabolic bariatric surgery, combined with our findings of exceptionally high preoperative eating disorder prevalence in Egyptian patients, suggests significant opportunity for postoperative psychological improvement. The core LABS consortium publications have established the methodological gold standards for longitudinal bariatric surgery research and eating disorder assessment protocols, providing the foundation for understanding these complex relationships [38].

While the Teen-LABS consortium’s work focuses on adolescent populations [39], their contributions to understanding eating disorder trajectories in younger bariatric patients inform our approach to comprehensive psychiatric evaluation. The convergence of our high prevalence findings with evidence of surgical therapeutic efficacy strongly supports the implementation of routine psychiatric screening in preoperative assessment, not only for risk stratification but also to establish baselines for measuring the substantial psychological benefits that may accompany successful bariatric intervention in this population.

The findings of this study fundamentally challenge current approaches to metabolic bariatric surgery candidacy by revealing that eating disorders, while dramatically more prevalent in Middle Eastern patients (47.8%) compared to Western populations (7.8%), represent significant therapeutic opportunities rather than contraindications to surgical intervention. Our results demonstrate a paradigm shift is needed: rather than viewing eating disorders as barriers to metabolic bariatric surgery, these patients constitute a high-yield population who may experience the greatest combined benefits of weight loss and psychological symptom improvement. The convergence of exceptionally high preoperative eating disorder prevalence with robust evidence from systematic reviews showing metabolic bariatric surgery as the most effective intervention for reducing eating disorder symptoms (SMD = 1.17) provides compelling rationale for integrated psychiatric care in bariatric surgery programs. This evidence-based approach transforms routine psychiatric screening from a gatekeeping mechanism to an essential tool for identifying patients who stand to benefit most from the dual therapeutic effects of surgical intervention, ultimately optimizing both physical and psychological outcomes in this high-need population while acknowledging the critical influence of cultural, socioeconomic, and regional factors on eating disorder presentation.

On the other hand, the prevalence of EDs in our study aligns with previous research findings. A study conducted in Iran found a much higher prevalence of eating disorders among MBS patients (77%) [28], while a Brazilian study reported a lower prevalence of 26.7% [40]. These discrepancies may be attributed to sociocultural differences, variations in sample size, and differences in diagnostic methods. Our study included a much larger sample (*N* = 3,240) compared to the Iranian (*N* = 284) and Brazilian (*N* = 281) studies, which likely enhanced the precision of our prevalence estimates. Additionally, while the Iranian study used the Longitudinal Assessment of Bariatric Surgery-2 (LABS-2) questionnaire and the Brazilian study relied on medical chart diagnoses, our study utilized SCID-5-RV, a structured diagnostic interview conducted by a trained psychiatrist.

BED prevalence differs from one study to another, as in the pattern of EDs. The prevalence of BED ranged from 90% in Aguiar et al.’s study [41] to 17% in Dawes et al.’s meta-analysis [42], with some studies reporting different prevalence values [28, 40]. NES and BN also have a discrepancy in their prevalence among the studies. In our study, NES was the second most common ED, followed by BN. In the Iranian study, BN was more common than NES (11% and 4%, respectively) [28], while in a Brazilian study, the same pattern was noted (6.6% BN and 5.3% NES) [40]. These variations in reported prevalence likely stem from several factors. Cultural differences play a significant role, including cultural attitudes toward body image, dietary patterns, and levels of stigma associated with mental health. Methodological differences also may have contributed to these discrepancies, including variations in assessment methods. Aguiar et al. [41] utilized the Binge Eating Scale, whereas Dawes’ meta-analysis [42] included studies employing various diagnostic tools, including structured clinical interviews.

The high prevalence of BED and NES in MBS patients highlights the need for comprehensive psychiatric assessments before surgery. Given that BED was significantly associated with depression and borderline personality disorder, targeted psychological interventions may be beneficial in addressing maladaptive eating behaviors before and after surgery [43]. These findings align with previous research demonstrating a strong association between depression and BED. Mitchell et al. [33] and Aguiar et al. [41] both found that depressive symptoms may increase the odds of BED, while Smith et al. [44] reported that atypical depressive disorders may increase the odds of BED tenfold. The relationship between depression and BED is likely driven by emotional eating as a coping mechanism for negative emotions, leading to excessive caloric intake and difficulties in weight regulation [45].

Although borderline personality disorder has not been widely studied in the literature for its association with BED, its core components, emotional dysregulation, and low self-esteem are core features of borderline personality disorder [46, 47] that have previously been associated with disordered eating behaviors. Mitchell et al. have found that emotional problems and lower self-esteem are correlated with BED [33]. Moreover, two recent meta-analyses found that lower self-esteem can predict EDs [48–50]. Emotional dysregulation may contribute to BED through maladaptive coping mechanisms, serving as a predictor for BED [33, 51]. The relationship between the components of borderline personality disorder and BED is intuitive. Individuals with emotional dysregulation often develop binge eating behaviors in the form of emotional eating as a maladaptive coping mechanism to manage negative emotions [52, 53]. Moreover, insecure attachment styles and avoidant or anxious attachment are associated with increased emotional dysregulation, leading to EDs [54]. Additionally, Self-esteem can lead to negative feelings towards one’s body, leading to BED. Also, self-esteem is a predictor of body image dissatisfaction in individuals seeking MBS, which may lead to emotional eating and EDs [55].

For the demographic variables and their association with BED, we found that the risk of BED slightly decreases with increasing age, a finding supported by community-based studies suggesting that BED tends to decline over time [56]. However, other studies have reported conflicting evidence, with some finding no significant association between age and BED [33, 51, 57]. The observed trend in our study may reflect the increased societal pressure on younger adults regarding body image and weight concerns [58], necessitating heightened awareness among clinicians treating younger MBS patients.

Females exhibited significantly higher odds of BED compared to males in our study (+ 67%). These results contradict the previous studies that found no association between gender and BED [40, 59]. Theoretically, women may have an increased risk of BED due to hormonal [60] or psychological factors [61]. Nevertheless, prospective studies, rather than cross-sectional studies, must adhere to this theoretical basis in future research.

Our study also found an inverse association between heavy smoking and BED, contradicting prior research that linked smoking with increased eating disorder risk [61–63]. One possible explanation is that smoking may serve as an appetite suppressant, masking binge eating behaviors rather than reducing their occurrence [63, 64]. However, given the well-documented adverse health effects of tobacco [65], this finding should be interpreted cautiously since moderate and no smoking was not significantly associated with BED. Future research should explore the complex interactions between smoking, appetite regulation, and binge eating tendencies.

The between-age and NES in our study (OR = 0.99) indicated a 1% reduction in the odds of experiencing NES for each advancing year. While certain prior studies reported no discernible relationship between age and NES [66, 67], others indicated a higher prevalence in younger adults, particularly those aged 18 to 30, with a notable decrease among individuals over 65 [68]. Our results are consistent with existing literature, demonstrating a statistically significant decline in NES odds among older populations.

In our study, the difference between females and NES was non-statistically significant. The literature revealed conflicting and inconclusive relations between gender and NES development. One study found a positive association between the male gender and NES [69]. Another study conducted in Saudi Arabia on post-metabolic-bariatric surgery patients found a much higher risk of NES in female patients (OR = 2.33, *P* = 0.03) [33]. However, other studies found no association between gender and NES [40, 68, 70]. Since females seek MBS more often, this can lead to spurious associations in studies focusing on MBS patients.

Heavy smoking was a significant predictor of NES, with heavy smokers exhibiting double the odds of NES compared to non-smokers (OR = 2.04, *P* < 0.001). This finding is consistent with prior research in both patients with obesity and the general population [71–73]. The link between smoking and NES appears to involve shared neurobiological pathways related to reward mechanisms and addiction [74, 75], alongside smoking’s function as a coping strategy for psychological distress [76, 77].

Moreover, depressive disorders were found to increase the likelihood of NES in our analysis, corroborating findings from earlier studies. A study reported a 38% increase in NES odds associated with depression [78]. Furthermore, depressive symptoms have been shown to modulate the nexus between food insecurity and NES, suggesting that depression is a significant factor in the onset of NES among patients for MBS [79, 80]. Moreover, studies have shown that depressive symptoms can lead to increased night eating, which exacerbates challenges in weight management and post-surgical outcomes [81, 82]. The co-occurrence of depressive symptoms, NES, and smoking further highlights the need for integrated treatment approaches addressing both behavioral and psychological aspects of NES.

Our cross-sectional study design was methodologically appropriate and provided several epidemiological advantages for addressing our research objectives. Cross-sectional studies represent the gold standard for prevalence estimation in epidemiological research, enabling the capture of point prevalence of eating disorders at the critical preoperative time point when clinical decision-making occurs. This design enabled us to establish baseline prevalence data for eating disorders in Egyptian bariatric surgery candidates - essential foundational epidemiological information that was previously unavailable for the MENA region. From an epidemiological perspective, prevalence studies serve as the cornerstone for understanding disease burden and informing public health interventions. The timing of our assessment is clinically relevant, as preoperative identification of eating disorders is crucial for treatment planning and risk stratification. While longitudinal studies offer valuable insights into causal relationships and temporal changes, our cross-sectional approach efficiently provided robust prevalence estimates using rigorous methodology with structured clinical interviews in a large, consecutive sample. The substantial prevalence we documented (47.8%) using this design has immediate clinical implications for preoperative screening protocols and supports the development of evidence-based guidelines for this population. Our epidemiological findings establish the necessary foundation for future longitudinal research, which should assess the impact of different preoperative eating disorders on postoperative weight loss and patient outcomes. These prospective studies should follow patients for sufficient duration, preferably more than 1 year, to avoid the “honeymoon” period in which the novelty and effectiveness of surgical intervention may temporarily mask or suppress unhealthy eating behaviors and problematic dietary patterns, thereby building upon our cross-sectional prevalence data to understand the natural history and causal pathways of eating disorders in this population.

### Limitations

The cross-sectional nature of this study limits our ability to draw causal conclusions about the relationships between psychiatric disorders and eating disorders since it is a snapshot in time. Although statistically significant associations were observed, the study design does not allow us to determine whether psychiatric disorders preceded the onset of eating disorders or vice versa. Furthermore, psychiatric disorders (excluding eating disorders) were identified through medical history rather than standardized psychometric evaluations, which might have led to misclassification bias. Our research focused solely on adult populations, limiting the generalizability of the findings to adolescents. Additionally, despite the study being conducted at a single center, the large sample size encompassing diverse regions across Egypt may enhance the representativeness and generalizability of the results. Given the sociocultural parallels between Egypt and other Arab countries [12], the findings may also have broader applicability across the Arab region.

## Conclusion

EDs are highly prevalent among MBS patients, with BED being the most common. Female gender, younger age, psychiatric disorders, and smoking habits are key predictors of EDs. These findings emphasize the need for routine psychiatric screening using validated assessment methods like the SCID-V to identify and support patients in an attempt to prevent recurrent weight gain and better understand the mechanism of potential suboptimal weight loss.

## Data Availability

The datasets used and analyzed during the current study are available from the corresponding author upon reasonable request.

## Abbreviations

AN: Anorexia Nervosa BED
BMI: Binge Eating Disorder
BMI: Body Mass Index
BN: Bulimia Nervosa
CI: Confidence Interval
DSM-5: Diagnostic and Statistical Manual of Mental Disorders Fifth Edition
EDs: Eating Disorders
LABS-2: Longitudinal Assessment of Bariatric Surgery-2
MBS: Metabolic Bariatric Surgery
MENA: Middle East and North Africa
NES: Night Eating Syndrome
OR: Odds Ratio
RWG: Recurrent Weight Gain
SCID-5: Structured Clinical Interview for DSM-5
